# Protocol for the development of a CONSORT extension for RCTs using cohorts and routinely collected health data

**DOI:** 10.1186/s41073-018-0053-3

**Published:** 2018-10-29

**Authors:** Linda Kwakkenbos, Edmund Juszczak, Lars G Hemkens, Margaret Sampson, Ole Fröbert, Clare Relton, Chris Gale, Merrick Zwarenstein, Sinéad M Langan, David Moher, Isabelle Boutron, Philippe Ravaud, Marion K Campbell, Kimberly A Mc Cord, Tjeerd P van Staa, Lehana Thabane, Rudolf Uher, Helena M Verkooijen, Eric I Benchimol, David Erlinge, Maureen Sauvé, David Torgerson, Brett D Thombs

**Affiliations:** 1 Behavioural Science Institute, Clinical Psychology, Radboud University, Nijmegen, the Netherlands; 2 NPEU Clinical Trials Unit, National Perinatal Epidemiology Unit, Nuffield Department of Population Health, University of Oxford, Oxford, UK; 3 Basel Institute for Clinical Epidemiology and Biostatistics, Department of Clinical Research, University Hospital Basel, University of Basel, Basel, Switzerland; 4 Library Services, Children’s Hospital of Eastern Ontario, Ottawa, Canada; 5 Department of Cardiology, Faculty of Health, Örebro University, Örebro, Sweden; 6 Centre for Clinical Trials and Methodology, Barts Institute of Population Health Science, Queen Mary University, London, UK; 7 Section of Neonatal Medicine, Department of Medicine, Imperial College London, Chelsea and Westminster Campus, London, UK; 8 Department of Family Medicine, Western University, London, Canada; 9 Institute for Clinical Evaluative Sciences, Toronto, Canada; 10 Faculty of Epidemiology and Population Health, London School of Hygiene and Tropical Medicine, London, UK; 11 Centre for Journalology, Clinical Epidemiology Program, Ottawa Hospital Research Institute, Ottawa, Canada; 12 INSERM, UMR1153, Paris, France; 13 Centre d’Épidémiologie Clinique, Hôpital Hôtel Dieu, Assistance Publique–Hôpitaux de Paris, Paris, France; 14 Faculté de Médecine, Université Paris Descartes, Sorbonne Paris Cité, Paris, France; 15 Health Services Research Unit, University of Aberdeen, Aberdeen, UK; 16 Health e-Research Centre, School of Health Sciences, Faculty of Biology, Medicine and Health, University of Manchester, Manchester, UK; 17 Faculty of Science, Division of Pharmacoepidemiology and Clinical Pharmacology, Utrecht University, Utrecht, the Netherlands; 18 Department of Health Research Methods, Evidence, and Impact, McMaster University, Hamilton, Canada; 19 Department of Psychiatry, Dalhousie University, Halifax, Canada; 20 University Medical Center Utrecht, Utrecht, the Netherlands; 21 University of Utrecht, Utrecht, the Netherlands; 22 Department of Pediatrics and School of Epidemiology and Public Health, University of Ottawa, Ottawa, Canada; 23 Institute for Clinical Evaluative Sciences, Ottawa, Canada; 24 Division of Gastroenterology, Hepatology and Nutrition, Children’s Hospital of Eastern Ontario, Ottawa, Canada; 25 Department of Cardiology, Clinical Sciences, Lund University, Lund, Sweden; 26 Scleroderma Society of Ontario, Hamilton, Canada; 27 Scleroderma Canada, Hamilton, Canada; 28 York Trials Unit, Department of Health Sciences, University of York, York, UK; 29 Lady Davis Institute for Medical Research, Jewish General Hospital, 4333 Cote Ste Catherine Road, Montreal, QC H3T 1E4 Canada; 30 Department of Psychiatry, McGill University, Montreal, Canada; 31 Department of Epidemiology, Biostatistics and Occupational Health, McGill University, Montreal, Canada; 32 Department of Medicine, McGill University, Montreal, Canada; 33 Department of Psychology, McGill University, Montreal, Canada; 34 Department of Educational and Counselling Psychology, McGill University, Montreal, Canada

**Keywords:** Administrative data, Cohort, CONSORT, Electronic health records, Electronic medical records, Electronic patient records, Randomized controlled trials, RCTs, Registries, Reporting guideline, Routinely collected health data

## Abstract

**Background:**

Randomized controlled trials (RCTs) are often complex and expensive to perform. Less than one third achieve planned recruitment targets, follow-up can be labor-intensive, and many have limited real-world generalizability. Designs for RCTs conducted using cohorts and routinely collected health data, including registries, electronic health records, and administrative databases, have been proposed to address these challenges and are being rapidly adopted. These designs, however, are relatively recent innovations, and published RCT reports often do not describe important aspects of their methodology in a standardized way. Our objective is to extend the Consolidated Standards of Reporting Trials (CONSORT) statement with a consensus-driven reporting guideline for RCTs using cohorts and routinely collected health data.

**Methods:**

The development of this CONSORT extension will consist of five phases. Phase 1 (completed) consisted of the project launch, including fundraising, the establishment of a research team, and development of a conceptual framework. In phase 2, a systematic review will be performed to identify publications (1) that describe methods or reporting considerations for RCTs conducted using cohorts and routinely collected health data or (2) that are protocols or report results from such RCTs. An initial “long list” of possible modifications to CONSORT checklist items and possible new items for the reporting guideline will be generated based on the Strengthening the Reporting of Observational Studies in Epidemiology (STROBE) and The REporting of studies Conducted using Observational Routinely-collected health Data (RECORD) statements. Additional possible modifications and new items will be identified based on the results of the systematic review. Phase 3 will consist of a three-round Delphi exercise with methods and content experts to evaluate the “long list” and generate a “short list” of key items. In phase 4, these items will serve as the basis for an in-person consensus meeting to finalize a core set of items to be included in the reporting guideline and checklist. Phase 5 will involve drafting the checklist and elaboration-explanation documents, and dissemination and implementation of the guideline.

**Discussion:**

Development of this CONSORT extension will contribute to more transparent reporting of RCTs conducted using cohorts and routinely collected health data.

## Background

The healthcare system is most effective when it is able to provide evidence-based care that is informed by research on the benefits and harms of interventions, as well as patients’ values and preferences. Randomized controlled trials (RCTs), when well-designed and conducted, are widely acknowledged to be the best design for providing such evidence [1–3]. However, important concerns about RCTs remain, including incomplete recruitment, small size, complex consent procedures, limited real-world generalizability, lack of relevant or long-term outcomes, and prohibitive costs [4–12].

The prohibitively high costs of conventional RCTs [13] have led to a declining number of them being conducted, and the majority of RCTs are conducted by industry with fewer investigator-initiated RCTs being performed [14]. At the same time, many interventions are adopted into practice despite a poor evidence base [15]. In response to these concerns, new approaches to RCTs have evolved that attempt to address issues related to logistics, cost, and relevance. These include RCT designs that utilize cohorts [5] and routinely collected health data to facilitate recruitment of participants and evaluate outcomes [16]. Routinely collected health data have been defined as data collected without specific a priori research questions developed prior to using the data for research [17]. Sources of routinely collected health data include registries [18, 19], electronic health records [20], and administrative databases [21]. Registries are set up for multiple purposes, including research, but most typically transfer routinely collected health data into a registry framework.

Cohorts and sources of routinely collected health data may differ in how participants are enrolled, how data are collected, and the degree of control that trial investigators have in determining available outcome measures. Nonetheless, the methodological principles of RCTs that are embedded in cohorts, registries, electronic health records, and administrative databases are similar. In cohorts, a group of individuals is gathered for the purpose of conducting research. Cohort-embedded RCTs use observational cohorts from which participants are subsequently enrolled in RCTs [22] or cohorts that are designed from inception to provide a framework for RCTs, as in the cohort multiple RCT (cmRCT) design [5]. In the cmRCT design, patients enroll in an observational cohort with regular outcome measurement. For each RCT embedded in the cohort, a random selection of RCT-eligible patients is contacted and offered access to the intervention. Outcomes of patients randomly allocated to the intervention group are compared to outcomes of RCT-eligible patients not randomly allocated to the intervention, who receive only usual care. Patients not selected are not notified that they are involved in the RCT usual care group, but their regularly collected cohort data are used to evaluate outcomes [5, 23]. A key aspect of the cmRCT design is that it replicates, as far as possible, consent practices that would be applied in clinical care, where patients are only notified about interventions that they can actually access. The number of studies that have implemented the cmRCT design is growing rapidly [24]. Examples include a recently completed RCT that involved treatment of patients with depressive symptoms from the UK South Yorkshire Cohort [25, 26]; a cohort of patients with the rare disease scleroderma, which will support RCTs of online rehabilitation, self-management, and psychological intervention programs [27]; a cohort for testing early interventions to prevent severe mental illness [28]; and cohorts of patients with cancer that will facilitate the conduct of RCTs of an exercise program [29, 30], radiation therapy [31, 32], and surgical interventions [33].

Disease or therapy registries are organized systems used to routinely collect patients’ health information for clinical, research, administration, or policy-related purposes [34]. Types of registries may include those that collect patient characteristics and outcome data for all patients with a specific disease or condition, all patients in a geographical region, or all patients who are treated within a health care service, or with a device or product. The type of data collected in registries varies, but may include a combination of data reported by patients, data recorded by health care providers, and data extracted from medical charts, institutional or organizational databases, biomaterial, and imaging data [35]. In registry-based RCTs, registries can be used for collection of baseline characteristics, to identify eligible patients for a RCT, and as the source of outcome data; with interactive methodology, they can also actively flag patients for enrollment in a RCT as patient data are entered in the registry [18, 19]. For example, the Swedish Coronary Angiography and Angioplasty Registry was used to enroll patients with ST-elevation myocardial infarction for an RCT of two different revascularization strategies [36]. Using the same registry, RCTs on invasive diagnostic strategies in coronary artery disease [37], different anticoagulation regimens in acute coronary syndrome [38], and the effect of oxygen therapy for patients with suspected myocardial infarction [39] have been conducted. In the USA, an RCT comparing post-procedure bleeding in percutaneous coronary intervention via radial versus femoral access was conducted within the National Institutes of Health National Cardiovascular Data Registry Cath-PCI Registry platform [40].

Electronic health records are electronic versions of a patient’s medical history in which data about all health care episodes are routinely collected. They can provide a method for comprehensive, often cross-institutional, longitudinal collection of patient health and health care data [41]. Electronic health records have been used previously to supplement data collection in RCTs [42], but are increasingly used to identify patients for RCT participation, assess them for eligibility, and assess outcomes using routinely collected data [43]. Electronic information systems in health care are becoming more common, and the richness and completeness of electronic health research databases have increased over time as more information is being shared electronically between different parts of the health-care system. An example of a RCT embedded in electronic health records involved a comparison of simvastatin and atorvastatin in patients with hypercholesterolemia and at high risk of cardiovascular disease [20]. Another example is an RCT that compared prophylactic with deferred or non-use of antibiotics in patients with mild to moderate exacerbation of chronic obstructive pulmonary disease. Both were conducted in the UK using routinely collected health records from general practitioners [20]. Electronic health records can also form part of an intervention to be tested in a clinical trial. For example, one RCT tested an intervention to reduce antibiotic prescribing by feeding back personalized antibiotic prescription data to primary care physicians [44]. Hence, clinical trials can incorporate electronic health record systems in multiple ways, at multiple stages and to very different degrees.

Health system administrative databases include information collected for administration or billing purposes (e.g., Medicare data in the USA). These data are routinely collected during clinic, hospital, laboratory, or pharmacy visits [45] and can provide easy and relatively inexpensive access to large numbers of patients over expansive geographic regions. They are increasingly being used for epidemiological, effectiveness, and safety outcomes research [45, 46]. As an example, in a cluster RCT that assessed whether printed educational messages would increase the use of thiazides as first-line medication for hypertension, a Canadian provincial administrative database was used to identify physicians with active general or family practices, and patient administrative data were used to assess patient outcomes [47]. Similar to electronic patient record systems, administrative databases can be used in RCTs at multiple points from recruitment, to forming part of the intervention, to data collection, and follow-up.

Because designs in which RCTs are embedded within cohorts and routinely collected health data reflect relatively recent innovations, published reports may not adequately describe important aspects of these RCTs. Reporting guidelines have been shown to improve the quality of reports [48–52], but there is currently no guideline for RCTs using cohorts and routinely collected health data. The CONsolidated Standards of Reporting Trials (CONSORT) reporting guideline, which includes a 25-item checklist and flow diagram, was developed to improve the quality of reporting of parallel group RCTs [53]. In recent years, several extensions of the CONSORT Statement have been developed to encourage better reporting of other types of trial designs, including, for instance, cluster trials [54], pilot and feasibility trials [55], and pragmatic trials [56].

RCTs embedded in cohorts and routinely collected health data share certain elements with “standard” RCTs covered in the CONSORT statement, but there are also aspects that differ and that may be important to report, including, for example:

- How participants were identified or selected and enrolled into the cohort or other external data collection system
- Details of information that was provided or not provided to participants about the potential for RCT participation upon entry
- How ethical approval and consent were handled at the observational data collection and RCT levels
- How RCT-eligible participants were identified and allocated to RCT arms
- Description of the host data collection system and markers of its quality or suitability for hosting a RCT
- Whether the availability of potential outcome measures in the external data collection system may have influenced the choice of RCT outcomes
- Whether definitions for data extraction were determined a priori
- How supplemental information such as the study protocol, raw data, or programming code can be obtained

Thus, it is possible that some items in the general CONSORT checklist may not be appropriate for reporting on RCTs using cohorts and routinely collected health data and may require modification; there may be other items that are essential for these types of RCTs, but are not included in the standard CONSORT statement.

Given the relative novelty of RCTs using cohorts and routinely collected health data, and the rapid pace at which these designs are being adopted, an extension of the CONSORT statement for these types of RCTs would be a timely means of improving long-term reporting quality by setting standards early in the process of uptake. This article describes the project plan for the development, testing, and dissemination of an extension of a CONSORT statement for RCTs conducted using cohorts and routinely collected health data.

## Methods

**Fig. 1:**
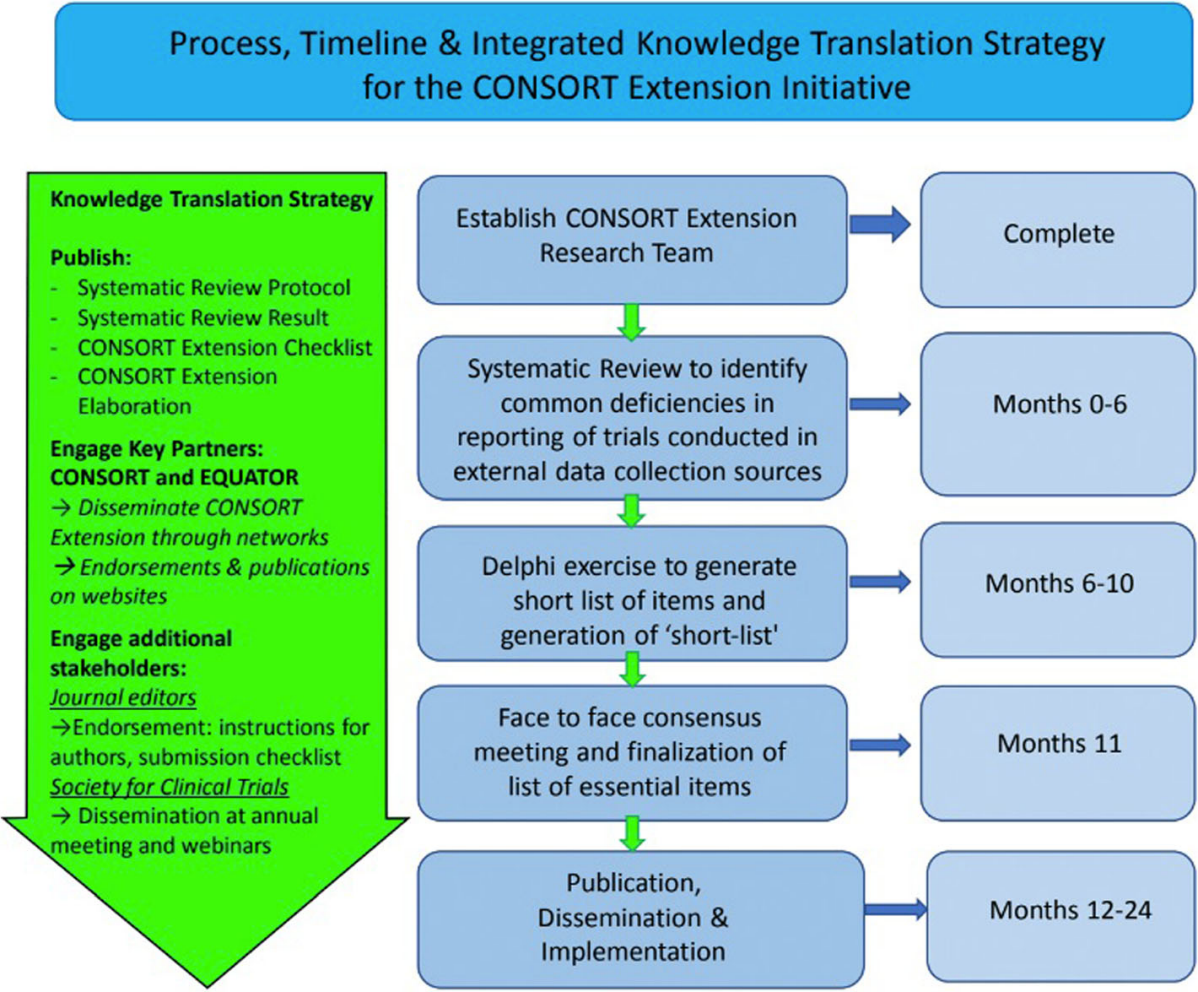
Summary of process, timeline, and knowledge translation strategy

The project has been registered with the Enhancing the QUAlity and Transparency Of health Research (EQUATOR) network [57]. This CONSORT extension will be developed using a sequential approach, consistent with previously published reporting guidelines created using the EQUATOR Network’s guidance [58]. It will consist of five phases. In phase 1, a research team and conceptual framework was established (completed in February 2018). In phase 2, we will generate a long list of items for this CONSORT extension from other key reporting guidelines and via a systematic review. In phase 3, this list will be prioritized using a Delphi exercise to assess appropriateness and importance of each potential item and to generate a shortlist of key items for consideration for inclusion. In phase 4, the shortlist of items will serve as the basis for an in-person consensus meeting to finalize a core set of items for inclusion in the reporting guideline and checklist. Phase 5 will involve write-up of the checklist and explanation and elaboration document, dissemination, and implementation. Figure 1 illustrates the planned process, timeline, and integrated knowledge translation approach.

### Phase 1: Research team and conceptual framework

To ensure project success, an organizational structure with a Project Team, a Steering Committee, and a group of key stakeholders has been defined.

#### Project team and steering committee

The project team is comprised of international experts in RCT methodology, including members who have designed and conducted RCTs using cohorts and routinely collected health data; experts in the development of reporting guidelines; experts in the conduct of systematic reviews; and experts in statistical methods for health research. A Steering Committee has been formed from team members to oversee aspects of the project, including database searching, study selection, data extraction, and data analysis, as well as the Delphi process, consensus meeting, write-up of the resultant guideline documents, and the implementation strategy. All team members will advise at each project stage and will help draft and disseminate the final guideline documents, help recruit stakeholders, and aid dissemination of the reporting guideline.

#### Knowledge users and stakeholders

Key stakeholders who will be involved throughout the process include guideline experts who disseminate guidelines to improve research (e.g., CONSORT, EQUATOR Network, Society for Clinical Trials); funders, health service decision-makers, journal editors; peer reviewers; trialists; and a patient representative who has been involved in engaging participants in RCTs embedded in a cohort. Knowledge users will advise the project team and be involved in dissemination and implementation.

### Phase 2: Systematic review and generation of long list of items

#### Preliminary “long list” generation

An initial “long list” of items will be formulated by reviewing the original CONSORT 2010 statement items [53]. We will then identify items for adaptation and areas where additional items may be needed by reviewing the Strengthening the Reporting of Observational Studies in Epidemiology (STROBE) [59] and REporting of studies Conducted using Observational Routinely collected Data (RECORD) [17] statements. These statements are considered the most relevant to this project because of their focus on reporting of observational studies and non-interventional studies using routinely collected health data. Additional “long list” items identified will be identified by our evidence review.

#### Registration of systematic review protocol

We will complete and publish our review protocol and describe the rationale, hypothesis, and planned methods according to the Preferred Reporting Items for Systematic reviews and Meta-Analyses for Protocols statement [60] and register it in PROSPERO (International Prospective Register of Ongoing Systematic Reviews, http://www.crd.york.ac.uk/prospero).

#### Database searches

Typically, reviews conducted for the development of reporting guidelines focus on articles about methods and reporting. Because of the novelty of RCTs using cohorts and routinely collected health data, we anticipate that we will identify only a small number of methods and reporting articles; thus, we will also review primary RCT protocols and reports of RCTs using these designs. We will review primary RCT reports to attempt to identify areas where reporting could be improved that may not be identified in the small number of existing methods articles that we anticipate being able to review.

In separate searches, we will seek publications that describe aspects of methods or reporting or that describe protocols or results from RCTs embedded in (1) cohorts; (2) registries; (3) electronic health records; and (4) administrative databases. MEDLINE strategies for the searches will be developed by a research librarian with input from the team and peer reviewed using the Peer Review of the Electronic Search Strategy standard [61]. The MEDLINE strategy will then be adapted for the Cochrane Library Methodology Register, which includes RCTs available up to its last update in July 2012. Search strategies will comply with Institute of Medicine standards and will not be limited by language [62]. We will search for examples of RCTs published in the last 10 years, which will allow us to identify relatively recent reporting practices and focus on challenging aspects of reporting. References of included studies will be hand-searched for additional eligible studies, and experts in the field will be consulted.

#### Study selection

For each search, separately, the search results will be downloaded into the citation management database RefWorks, and duplicate references will be removed. Following this, references will be transferred into the systematic review software DistillerSR®. A coding manual based on eligibility criteria will be developed, and a pilot test of the coding manual will be performed. Using the coding manual, we will assess the eligibility of each publication through a two-stage process. First, using a liberal accelerated approach [63], we will screen titles and abstracts. Articles will be included in the full-text review stage if one reviewer deems them potentially eligible, but two reviewers will be needed to exclude an article. Then a full-text review will be completed, with two investigators independently reviewing each article. Disagreements after full-text review will be resolved by consensus, with a third investigator consulted as necessary. If the number of eligible articles identified is large, we will select a random sample for review and data extraction.

#### Data extraction and verification

Eligible articles will be reviewed by the team to create a “long list” of items as follows: for publications that describe aspects of methods or reporting of RCTs conducted using cohorts and routinely collected health data, we will review the publications and identify elements of RCT design that are important to report. Potential items should clarify or modify an existing CONSORT 2010, STROBE, or RECORD item or if they suggest a new element that should be reported as a separate item. Elements will be checked for redundancy between investigators, and if either investigator or both deem it important to report, the item will be included in the long list. For publications describing protocols or results from RCTs using cohorts and routinely collected health data, two investigators will independently review publications to identify potential modifications or clarifications of CONSORT 2010, STROBE, and RECORD items and to identify potential additional items. All identified items will be added to the initial long list of items (with removal of duplicates). Data will be collected in DistillerSR® using a standardized data extraction form.

#### Long list verification

Members of the Steering Committee will assess each item on the long list to exclude items that are clearly not relevant, prior to starting the Delphi process. Each assessor will independently evaluate the long-list of items and will score potential items as “potentially relevant” or “clearly not relevant.” Items that are assessed as “clearly not relevant” by all members of the Steering Committee will be removed. Items identified as potentially relevant by any of the members will be included on the long list of items for evaluation in the Delphi exercise.

### Phase 3: Delphi exercise and generation of “short list”

The “long list” created in phase 2 will serve as a base for a three-round Delphi exercise that will be conducted using established guidance for health care applications [64, 65]. This Delphi process has been used previously for similar work in development of reporting guidelines [66].

The aim of this process is to achieve consensus on essential items to be included in this CONSORT extension. The Delphi process will be facilitated by team member with experience in the Delphi process and in guideline development. The Delphi participants will include all project team members and knowledge users; additional participants may be added if they are identified as potential experts during the systematic review phase (e.g., authors of published RCTs using cohorts and routinely collected health data). There is no agreement on the minimum or “ideal” panel size for Delphi studies, but we will aim for a minimum of 20 participants, as this has been suggested to provide stable results [67].

For each survey round, participants will be invited by email, and they will have 2 weeks to complete each survey online (with a reminder 1 week prior to survey closure). All participants will be invited to each round of the survey regardless of whether they completed the previous round.

Starting with the long list of potential items from phase 2, during the survey, participants will be asked to rank items based on how essential they are for reporting of RCTs embedded in cohorts and routinely collected health data on a 1–5 Likert scale (1 = not essential; 5 = essential). Likert scores will be categorized as follows: 1–2 = low score (item should not be part of CONSORT extension checklist), 3 = moderate (item should be discussed), 4–5 = high score (item should be part of CONSORT extension checklist). Participants who rank items will also be able to provide comments via an open-comment box. When any item is ranked in the same category (i.e., low, moderate, or high) by more than 2/3 of the Delphi respondents it will be deemed to meet “consensus”; items that reach consensus in a Delphi round will be removed from the following Delphi round [67]. Participants will also be asked to suggest any additional potentially relevant items and provide a brief rationale and clarification for the item.

The second round of the survey will include any items that did not reach consensus in the first round and any new items suggested by respondents in the first round. Results of the first round will be summarized (i.e., for each item, the number of participants who have scored the item and the distribution of scores, as well as a synthesis of comments on the item) and will be fed back anonymously to Delphi respondents. Respondents will be invited to review their initial responses, considering the responses from other Delphi respondents, and to re-score the item on those tasks not achieving consensus.

As with the second round, the third round will involve items that did not reach consensus in the first or second round and any newly suggested items. In the third round, the results from the second round will again be fed back to all respondents, who will again be asked to rate each item.

Following the three rounds, the mean and median scores for each item will be tabulated, and items will be categorized as follows: no consensus (< 2/3 participants rate the item in the category); consensus is reached for a rating of moderate (3); consensus is reached for an item rated as low (1, 2); consensus is reached for an item rated as high (4, 5). Results of the Delphi exercise will be presented during the face-to-face meeting to help inform the selection of checklist items.

### Phase 4: Face-to-face consensus meeting and finalization of list of essential items

A 2-day consensus meeting will be organized, and members of the project team as well as key stakeholders will be invited to participate. The main aim of this meeting will be to reach consensus on items, informed by the Delphi survey, and to arrive at a final list of essential items to report on RCTs using cohorts and routinely collected health data. The process will involve presentation of items by attendees with key expertise followed by discussion. Items that reached consensus in the Delphi survey will be considered first in order to ratify those results. Each remaining item will then be considered, and meeting participants will be given an opportunity to discuss each item, followed by anonymous scoring by those at the consensus meeting. Meeting participants will also be able to bring forward items excluded during the Delphi process for reconsideration if they believe that a clearer articulation or better explanation of the item might alter the team’s conclusion. We will attempt to arrive at consensus among meeting participants, and voting will be implemented if no consensus can be reached.

It is anticipated that some items will be applicable for all types of RCTs conducted via cohorts and routinely collected health data, whereas some may not apply to all designs, and this will be clarified. Additional aims of this meeting include reviewing the CONSORT flow diagram to determine if any revisions are necessary, discussing an outline of the checklist and elaboration-explanation document, and planning the publication and dissemination strategy.

### Phase 5: Knowledge translation

#### Checklist pilot, revision of checklist based on feedback, and guidance statements

After finalizing the checklist at the consensus meeting, the team will apply the checklist to a random sample of published reports that describe results from relevant RCTs in order to identify practical challenges and to inform writing of the statement and explanation and elaboration. The piloting phase will include testing by members of the research team, other trialists who will be invited to pilot the checklist, and graduate students from participating institutions who are enrolled in trial-related courses.

Steering committee members will lead the writing of the final version of the checklist and the associated guidance statement. This will be circulated to the broader project team for input prior to submission for publication. Members of the steering committee will also lead the writing of the explanation and elaboration document, which will serve as a “user guide” for the checklist and will include an item-by-item explanation plus examples of good reporting practice to aid interpretation. This explanatory document will also be circulated to the broader research team for input prior to submission for publication.

#### Baseline assessment

A study with the aim to provide a baseline assessment of completeness of reporting of RCTs using cohorts and routinely collected health data prior to publication of the reporting guideline, as measured by this CONSORT extension checklist, will be conducted in order to track change in reporting quality over time.

#### Dissemination

A detailed knowledge transfer plan will be developed at the face-to-face meeting in phase 4 using strategies that have been successfully implemented by EQUATOR for similar reporting guidelines. Multiple simultaneous publications of the CONSORT extension checklist and the explanation and elaboration document will be sought in journals that may publish RCTs that use cohorts and routinely collected health data. Further dissemination will include presentation at major medical, health care policy and trial-related conferences, presentation at editorial forums and to major funders of RCTs, publication on the CONSORT and EQUATOR websites, and dissemination through relevant social media channels.

#### Implementation

Activities aimed at user training and encouraging uptake of this CONSORT extension will include implementation through strategies that have been used successfully previously in similar projects by knowledge user partners from CONSORT and EQUATOR (e.g., workshops and lectures), guided by members of the project team. We will also reach out to journal editors to encourage endorsement and implementation of this CONSORT extension for RCTs using cohorts and routinely collected health data. This could be in the form of including this CONSORT extension in the instructions for authors as a source of guidance and submission of the CONSORT extension checklist with the manuscript. Other endorsement and implementation strategies, as well as a strategy for evaluating effectiveness will be discussed during the face-to-face meeting.

## Discussion

Development of this CONSORT extension for RCTs conducted using cohorts and routinely collected health data is being done with the goals of contributing to transparent reporting of these types of RCTs, reducing research waste, and facilitating their reproducibility.

## Abbreviations

cmRCT: Cohort multiple randomized controlled trial
CONSORT: COnsolidated Standards of Reporting Trials statement
EQUATOR: Enhancing the QUAlity and Transparency Of health Research
PROSPERO: International Prospective Register of Ongoing Systematic Reviews
RCT: Randomized controlled trial
RECORD: REporting of studies Conducted using Observational Routinely-collected health Data statement
STROBE: Strengthening the Reporting of Observational Studies in Epidemiology statement
